# Modification of intracellular glutathione status does not change the cardiac trapping of ^64^Cu(ATSM)

**DOI:** 10.1186/s13550-014-0040-8

**Published:** 2014-08-01

**Authors:** Fiona Shaughnessy, Erika Mariotti, Karen P Shaw, Thomas R Eykyn, Philip J Blower, Richard Siow, Richard Southworth

**Affiliations:** Division of Imaging Sciences, The Rayne Institute, St. Thomas’ Hospital, King’s College London, Lambeth Palace Rd., London, SE1 7EH UK; Cardiovascular Division, Franklin-Wilkins Building, King’s College London, Waterloo Campus, 150 Stamford St., London, SE1 9NH UK

**Keywords:** 64Cu(ATSM), bis(thiosemicarbazones), Hypoxia imaging, Glutathione

## Abstract

**Background:**

The trapping mechanisms of the PET hypoxia imaging agent copper(II)-diacetyl-*bis*(*N*^4^-methylthiosemicarbazone) (^64^Cu(ATSM)) remain unresolved, although its reduction prior to dissociation may be mediated by intracellular thiols. Glutathione (GSH) is the most abundant intracellular thiol, and its redox status changes in cancer cells and ischaemic myocardium (two prime applications for ^64^Cu(ATSM) PET). We therefore investigated whether modification of intracellular GSH content affects the hypoxia selectivity of ^64^Cu(ATSM).

**Methods:**

Isolated rat hearts (*n* = five per group) were perfused with aerobic buffer (equilibrated with 95%O_2_/5%CO_2_) for 15 min, then hypoxic buffer (95%N_2_/5%CO_2_) for 20 min. Cardiac glutathione was depleted by buthionine sulphoximine (BSO, 4 mmol/kg/ 48 h intraperitoneal), or augmented by *N*-acetyl cysteine (NAC, 4 mmol/L) in the perfusion buffer. Cardiac ^64^Cu retention from three 2-MBq bolus injections of ^64^Cu(ATSM) before and during hypoxia was then monitored by NaI detectors.

**Results:**

Cardiac GSH content was elevated by NAC and depleted by BSO (from 7.9 ± 2.0 to 59.3 ± 8.3 nmol/mg and 3.7 ± 1.0 nmol/mg protein, respectively; *p* < 0.05). Hypoxia did not affect cardiac GSH content in any group. During normoxia, tracer washed out bi-exponentially, with 13.1% ± 1.7% injected dose being retained; this was not affected by GSH augmentation or depletion. Hypoxia significantly increased tracer retention (to 59.1% ± 6.3%, *p* < 0.05); this effect was not modified by GSH augmentation or depletion.

**Conclusion:**

Modification of GSH levels had no impact upon the pharmacokinetics or hypoxia selectivity of ^64^Cu(ATSM). While thiols may yet prove essential for the intracellular trapping of ^64^Cu(ATSM), they are not the determinants of its hypoxia selectivity.

**Electronic supplementary material:**

The online version of this article (doi:10.1186/s13550-014-0040-8) contains supplementary material, which is available to authorized users.

## Background

Radiocopper *bis*(thiosemicarbazone) complexes demonstrate promise as hypoxia imaging agents, with potential applications in oncology and cardiology. The lead compound in this class, copper(II)-diacetyl-*bis*(*N*^4^-methylthiosemicarbazone) (^64^Cu(ATSM)) has previously been demonstrated to identify hypoxic regions in tumours [1]–[4] and the myocardium [5]–[8] with high first pass uptake and rapid clearance from normoxic tissue. The mechanism of its tissue retention, however, remains unclear [9],[10]. It is currently thought that the lipophilic complex, Cu(II)(ATSM), diffuses into the cell, whereupon it is reduced intracellularly to Cu(I)-ATSM^−^[9],[10]. Under normoxic conditions, this unstable Cu(I)(ATSM)^−^ species is reoxidised to Cu(II)(ATSM) and leaves the cell. In hypoxic tissue, however, there is insufficient oxygen to reoxidise Cu(I)(ATSM), which dissociates, releasing its radiocopper core to become trapped within the cell by copper sequestering proteins. Although it is apparent that hypoxia promotes radiocopper retention, the reductants responsible for the initial reduction remain unidentified.

Early *in vitro* experiments demonstrated that thiols were capable of reducing 3-ethoxy-2-oxobutyraldehyde *bis*(thiosemicarbazone) (Cu-KTS), leading to the suggestion that intracellular thiols may be responsible for the reduction of these complexes [11],[12]. The antioxidant glutathione (GSH) is the most abundant thiol-containing species intracellularly, at an average intracellular concentration of approximately 5 mmol/L [13]. Since intracellular GSH status changes in numerous diseases (particularly cardiac ischaemia and cancer [14]–[16]), such variation in intracellular GSH-mediated reductive capacity may impact upon ^64^Cu(ATSM) hypoxia selectivity and its potential clinical usefulness.

We have recently described an isolated perfused heart system coupled with a triple NaI gamma detection apparatus which allows the characterisation of radiotracer pharmacokinetics in an intact functioning organ over which we have complete functional control [8],[17]. This approach allows us to perform reproducible interventions (such as accurately titrated hypoxia) under conditions which may otherwise be lethal *in vivo* and examine tracer pharmacokinetics in a tissue of interest directly, without the added complications of circulating tracer metabolites which can frequently be problematic *in vivo*. In this study, we have utilised this approach to determine whether depletion or augmentation of intracellular GSH status impacts upon the pharmacokinetics or hypoxia selectivity of ^64^Cu-ATSM.

## Methods

### Chemicals and reagents

dl-buthionine (*S*,*R*)-sulphoximine 99% (BSO), trichloroacetic acid 6.5% and the bicinchoninic acid (BCA) assay kit were obtained from Fisher Scientific (Loughborough, UK). *N*-acetyl-l-cysteine 99% (NAC) and *O*-phthalaldehyde (OPA) were obtained from Sigma-Aldrich (Dorset, UK).

### ^64^Cu(ATSM) synthesis

^64^Cu was provided by the PET imaging centre, St. Thomas' Hospital, London. ATSM was labelled with ^64^Cu as described by Handley et al. [18].

### Animals

Male Wistar rats (275 to 350 g) with *ad libitum* access to food and water were used throughout. All experimental procedures were carried out in accordance with Home Office regulations as detailed in the Guidance on the Operation of Animals (Scientific Procedures) Act 1986.

### GSH depletion/augmentation

GSH levels were depleted by pre-treating rats twice daily for 2 days with intraperitoneal injections of BSO dissolved in 0.9% NaCl (total dose 4 mmol/kg body weight). Hearts were excised and Langendorff-perfused 24 h after the final dose. In further groups of animals, the cardiac GSH concentration was augmented by supplementing the perfusion medium, (modified Krebs Henseleit buffer (KHB), with NAC (4 mmol/L).

### Heart perfusion

Rats were anaesthetised with sodium pentobarbitone and heparinised (200 IU intraperitoneal). The hearts were excised and placed immediately in KHB (4°C) comprising NaCl (118 mmol/L), KCl (5.9 mmol/L), MgSO_4_ (1.16 mmol/L), NaHCO_3_ (25 mmol/L), NaEDTA (0.48 mmol/L), glucose (11.1 mmol/L) and CaCl_2_ (2.2 mmol/L) and then Langendorff-perfused at a constant rate of 14 mL/min with KHB gassed with 95%O_2_/5%CO_2_ at 37°C. To induce cardiac hypoxia, perfusion was switched to KHB gassed with 95%N_2_/5%CO_2_. Buffer oxygen saturation was monitored throughout each experiment by an OxyLite™ fluorescent oxygen probe (Oxford Optronix Ltd., Oxfordshire, UK) inserted into the arterial perfusion line. Coronary perfusion pressure was monitored via a pressure transducer mounted in the arterial line. Cardiac contractile function was monitored via a pressure transducer connected to a latex balloon inserted into the left ventricle, inflated to give an end-diastolic pressure of 4 to 9 mmHg. Coronary effluent was collected at regular intervals and subsequently analysed for lactate content using a 2300 STAT Plus™ lactate analyser (YSI Ltd., Hampshire, UK).

### Perfusion protocols

**Figure 1:**
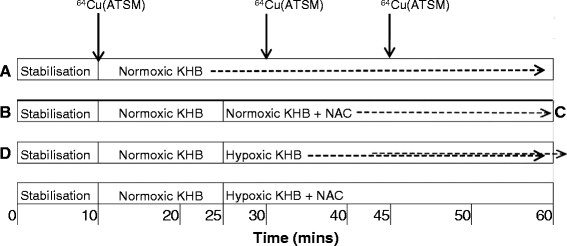
**Perfusion protocols.** Protocols are for hearts from all treatment groups and show timings of ^64^Cu(ATSM) bolus administration (arrows). **(A)** Normoxic control with/without GSH depletion, **(B)** normoxic GSH augmented, **(C)** hypoxic control with/without GSH depletion and **(D)** hypoxic GSH augmented groups.

All hearts were perfused with normoxic KHB for a stabilisation period of 10 min to ensure cardiac contractile function exclusion criteria were met before continuing each experiment. The hearts were then perfused for a further 45 min according to the protocols in Figure 1. Three boluses of ^64^Cu(ATSM) (2 MBq in 100 μL KHB) were injected into the arterial perfusion line after 10-min normoxic perfusion and 5 and 25 min after the onset of hypoxia (or normoxic equivalent). A custom-built triple detector system was used to measure cardiac ^64^Cu retention and washout [8]. This comprised three orthogonally arranged lead collimated Na/I γ-radiation detectors (Raytest Isotopenmessgeräte GmbH, Straubenhardt, Germany) measuring ^64^Cu activity at the input (arterial) perfusion line, the heart and the output perfusion line. The detectors were connected to a Gina Star™ data acquisition system (Raytest Isotopenmessgeräte GmbH), and data were acquired by Gina Star™ software (version 4.0.2.75).

Data were normalised to the maximum peak counts after each injection and corrected for decay and cardiac background activity 30 s prior to each injection [8]. Pharmacokinetic analysis of time activity curve data was performed using MATLAB® (version 7.11.0, MathWorks®, Natick, MA, USA) and fitted with a bi-exponential function:

1 $$f\left(t\right)=ae^{-\mathrm{bt}}+ce^{-\mathrm{dt}}$$

where *b* and *d* represent the slow and fast clearance rate constants (SCR and FCR), and *a* and *c* are the amplitudes assigned to these constants, respectively, as described previously [8],[17],[19].

### GSH measurement in heart tissue

At the end of each perfusion protocol, the hearts were snap-frozen in liquid nitrogen and stored at −70°C. The hearts were ground into a fine powder under liquid nitrogen using a steel pestle and mortar. Of this powder, 0.5 g was weighed into centrifuge tubes, and thiols were extracted by adding 2.5 mL ice-cold trichloroacetic acid for 20 min. The samples were centrifuged at 10,000 rpm at 4°C for 10 min. The trichloroacetic acid supernatant was then aspirated and analysed for GSH content using the OPA fluorescence assay as described previously [20]. NaOH (2.5 mL, 1 mmol/L) was then added to the cell pellet for 2 h, then aspirated and analysed for protein content using a BCA assay kit [21].

### Statistical analysis

All data are presented as the mean ± standard deviation. Statistical significance was evaluated using a one-way ANOVA followed by Bonferroni *post hoc* test using GraphPad Prism (GraphPad Software Inc., San Diego, CA, USA).

## Results

### Effect of BSO and NAC on myocardial GSH concentration

**Figure 2:**
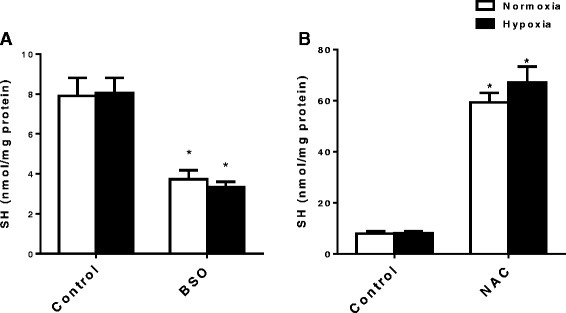
**Effect of (A) BSO pre-treatment and (B) NAC perfusion on myocardial GSH (SH) content.** Data are expressed as means ± SD (*n* = 5 per group). *p < 0.05 vs. untreated equivalents.

BSO pre-treatment caused a significant depletion of GSH concentration (from 7.9 ± 2.0 to 3.7 ± 1.0 nmol/mg protein, *p* < 0.05, Figure 2). These values were not affected by perfusion with hypoxic buffer (8.0 ± 1.7 and 3.3 ± 0.6 nmol/mg protein). Perfusion with NAC caused a significant increase in GSH concentration (from 7.9 ± 2.0 to 59.3 ± 8.3 nmol/mg protein, *p* < 0.05), and these values were also unaffected by perfusion with hypoxic buffer (8.0 ± 1.7 and 67.1 ± 13.9 nmol/mg protein). The perfusion of hearts with hypoxic buffer had no effect on cardiac GSH status in any group.

### Myocardial uptake of ^64^Cu from ^64^Cu(ATSM) during normoxia and hypoxia

**Figure 3:**
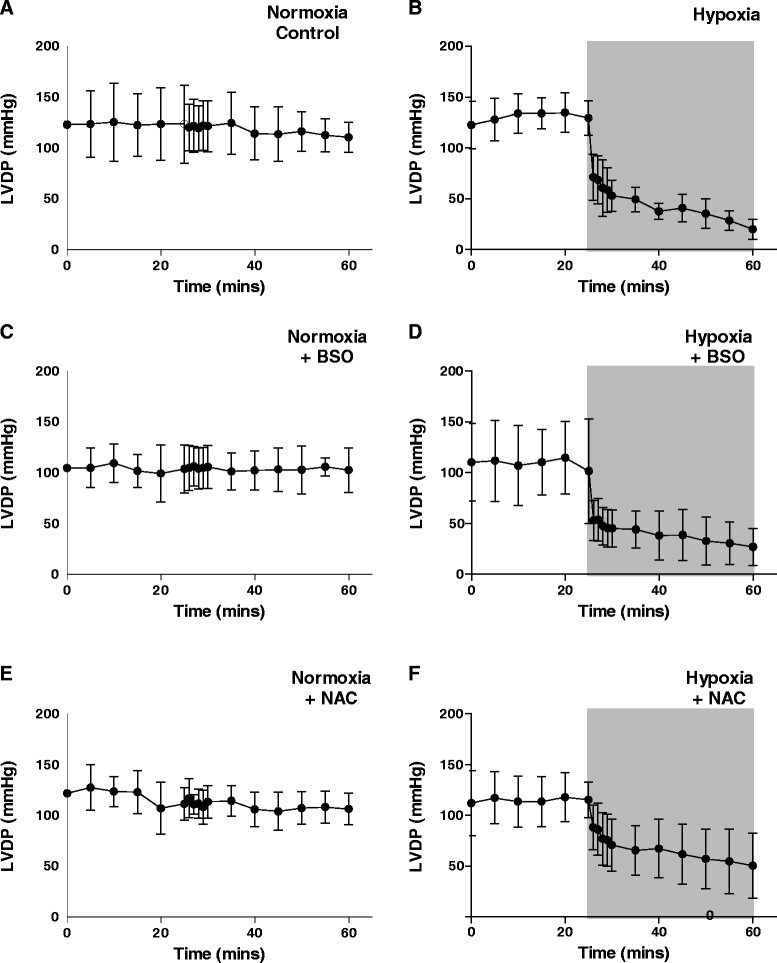
**Left ventricular developed pressure (mmHg) for hearts from all perfusion protocol groups.** Data are expressed as means ± SD (*n* = 5). **(A)** Normoxia control, **(B)** hypoxia, **(C)** normoxia + BSO, **(D)** hypoxia + BSO, **(E)** normoxia + NAC, **(F)** hypoxia + NAC.

**Figure 4:**
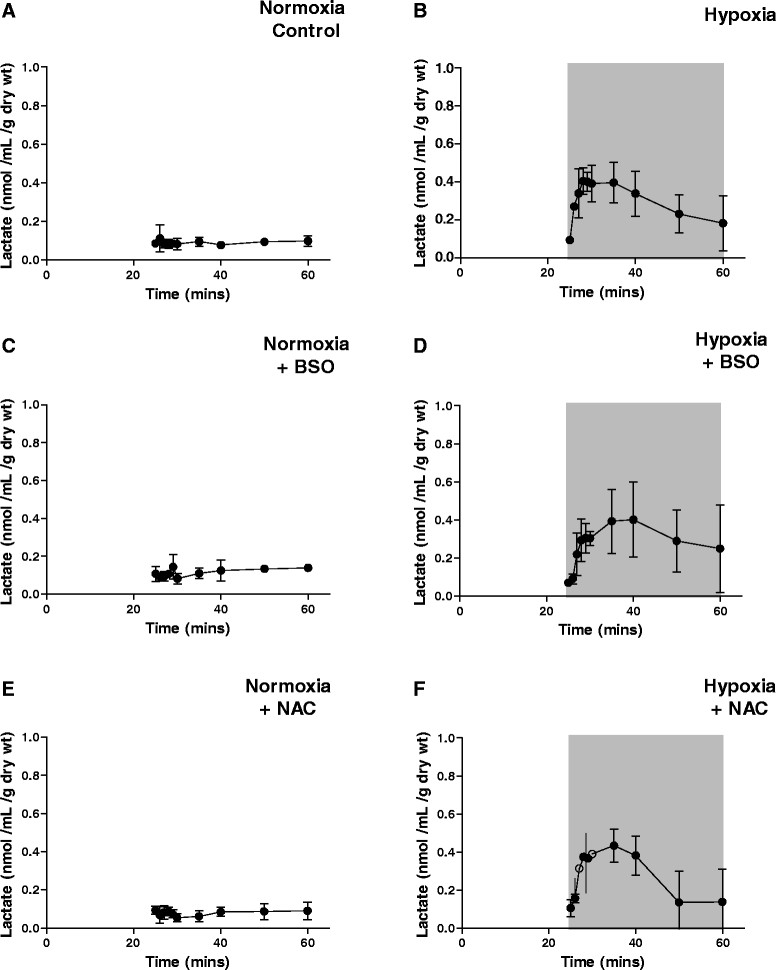
**Cardiac lactate release.** Lactate concentration was measured in perfusate samples collected from isolated perfused hearts. Data are expressed as means ± SD (*n* = 5). **(A)** Normoxia control, **(B)** hypoxia, **(C)** normoxia + BSO, **(D)** hypoxia + BSO, **(E)** normoxia + NAC, **(F)** hypoxia + NAC.

**Figure 5:**
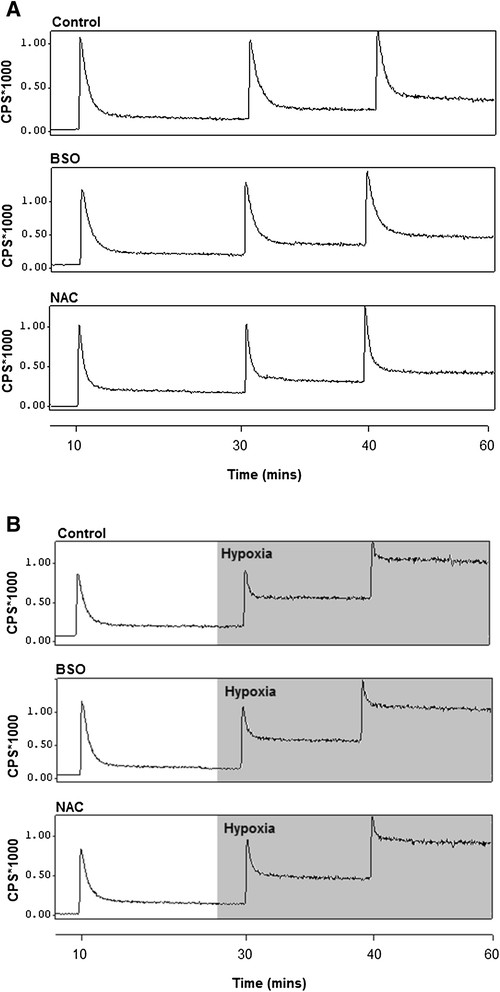
**Representative time-activity curves demonstrating**^**64**^**Cu retention from**^**64**^**Cu(ATSM) in (A) normoxic and (B) hypoxic hearts.** Curves represent untreated hearts (top), GSH depleted hearts (centre) and GSH augmented hearts (bottom).

**Figure 6:**
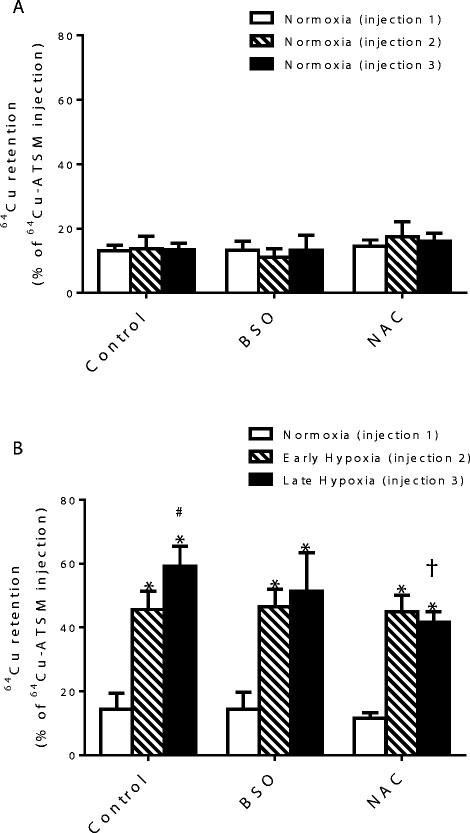
**Effect of GSH concentration on cardiac**^**64**^**Cu retention from**^**64**^**Cu(ATSM).**^64^Cu retention was measured in GSH depleted (BSO) and augmented (NAC) hearts during **(A)** normoxia and **(B)** hypoxia. Data are expressed as the percentage of total activity injected (means ± SD, *n* = 5 per group). *p < 0.05 vs. normoxia (injection 1), #*p* < 0.05 vs. early hypoxia (injection 2) and †*p* < 0.05 vs. late hypoxia alone (injection 3).

Buffer oxygen saturation at the perfusion cannula fell from >100 to <20 mmHg within 5 min and to <5 mmHg within 20 min of switching to hypoxic buffer, and left ventricular developed pressure fell from 129.3 ± 16.8 to 53.0 ± 15.3 mmHg within 5 min to 0 mmHg after 20 min (Figure 3) [8]. Lactate release peaked at 0.4 ± 0.07 nmol/mL dry weight tissue after 3 min of switching to hypoxic buffer, before declining as contractility decreased (Figure 4). Neither GSH depletion nor augmentation affected developed pressure or lactate release during perfusion with normoxic or hypoxic buffer. Figure 5 displays representative traces from the second Na/I detector, monitoring cardiac ^64^Cu radiotracer uptake and washout. During normoxic perfusion, 13.1% ± 1.7% of the injected dose (ID) remained in the heart 20 min after the first injection. This did not significantly differ in subsequent injections (Figure 6A). Neither GSH augmentation nor GSH depletion had any effect on cardiac ^64^Cu retention during normoxic perfusion. Perfusion of hearts with hypoxic buffer caused a significant increase in tracer retention to 45.6% ± 5.8% ID after 5 min and further still to 59.1% ± 6.3% ID after 25 min (Figure 6B). Neither depletion nor augmentation of GSH affected this hypoxia-dependent retention (11.2% ± 2.6% to 13.2% ± 4.6% in GSH-depleted and 46.5% ± 5.5% to 51.3% ± 12.1% in GSH-augmented hearts).

### ^64^Cu(ATSM) pharmacokinetics

Using non-linear spectral analysis, we determined that cardiac tracer washout was biphasic, with fast and slow components [17]. The FCR (*d*) of tracer from normoxic hearts was consistent throughout the perfusion protocol and was unaffected by either GSH augmentation or depletion. There was no difference in FCR in hearts from normoxic control, GSH-depleted or GSH-augmented protocols across all injections (Table 1). Similarly, there was no difference in SCR from these hearts. The amplitude of the FCR and SCR, *c* and *a* respectively, also did not differ between different injections or different treatment groups under normoxic conditions (Table 2). The *c* values were significantly higher than *a* in all hearts under normoxic conditions.

**Table 1:** **Effect of GSH concentration on the fast and slow clearance rates of** ^**64**^ **Cu(ATSM)**

Clearance rate	Type of heart	Injection	Untreated	BSO	NAC
FCR (*k*_d_ (min^−1^))	Normoxic hearts	Normoxia	1.2 ± 0.2	1.1 ± 0.1	1.3 ± 0.2
		Normoxia	1.1 ± 0.2	1.1 ± 0.2	1.4 ± 0.1
		Normoxia	1.2 ± 0.1	1.2 ± 0.2	1.4 ± 0.4
	Hypoxic hearts	Normoxia	1.2 ± 0.2	1.1 ± 0.2	1.2 ± 0.2
		Early hypoxia	1.9 ± 0.4	1.8 ± 0.5	1.9 ± 0.3
		Late hypoxia	2.5 ± 0.6*	2.2 ± 0.5*	2.2 ± 0.8*
SCR (*k*_b_ (min^−1^))	Normoxic hearts	Normoxia	0.01. ± 0.002	0.008 ± 0.002	0.01 ± 0.006
		Normoxia	0.01 ± 0.002	0.01 ± 0.008	0.01 ± 0.006
		Normoxia	0.01 ± 0.002	0.01 ± 0.004	0.01 ± 0.004
	Hypoxic hearts	Normoxia	0.01 ± 0.007	0.01 ± 0.002	0.01 ± 0.002
		Early hypoxia	0.007 ± 0.005	0.008 ± 0.005	0.009 ± 0.002
		Late hypoxia	0.003 ± 0.003*	0.004 ± 0.002*	0.005 ± 0.002*

The effect of GSH supplementation (with NAC) and GSH depletion (with BSO) upon the fast clearance rate (FCR) and slow clearance rate (SCR) of ^64^Cu(ATSM) in normoxic and hypoxic hearts. Data represent means ± SD, *n* = 5 per group, *p < 0.05 vs. normoxic equivalent.

**Table 2:** **Effect of GSH concentration on the amplitudes of fast and slow clearance rates of** ^**64**^ **Cu(ATSM)**

Amplitude of clearance rate	Type of heart	Injection	Untreated	BSO	NAC
FCR	Normoxic hearts	Normoxia	0.7 ± 0.02	0.8 ± 0.07	0.7 ± 0.07
		Normoxia	0.6 ± 0.04	0.7 ± 0.07	0.6 ± 0.04
		Normoxia	0.6 ± 0.04	0.6 ± 0.1	0.6 ± 0.06
	Hypoxic hearts	Normoxia	0.8 ± 0.04	0.8 ± 00.07	0.8 ± 0.04
		Early hypoxia	0.4 ± 0.06	0.4 ± 0.06	0.4 ± 0.05
		Late hypoxia	0.2 ± 0.03	0.2 ± 0.06	0.2 ± 0.03
SCR	Normoxic hearts	Normoxia	0.2 ± 0.03*	0.2 ± 0.04*	0.2 ± 0.03*
		Normoxia	0.3 ± 0.04*	0.2 ± 0.04*	0.3 ± 0.05*
		Normoxia	0.3 ± 0.03*	0.3 ± 0.05*	0.4 ± 0.04*
	Hypoxic hearts	Normoxia	0.2 ± 0.04*	0.2 ± 0.04*	0.2 ± 0.02*
		Early hypoxia	0.5 ± 0.1	0.5 ± 0.02	0.5 ± 0.06
		Late hypoxia	0.7 ± 0.05*	0.7 ± 0.05*	0.7 ± 0.05*

The effect of GSH supplementation (with NAC) and GSH depletion (with BSO) upon the amplitudes of the fast clearance rate (FCR) and slow clearance rate (SCR) of ^64^Cu(ATSM) in normoxic and hypoxic hearts. Data represent means ± SD, *n* = 5 per group, *p < 0.05 vs. FCR.

Hypoxia caused a progressive increase in the FCR, and a decrease in the SCR, becoming significantly different from normoxic values in all groups by 25 min of hypoxia (Table 1). None of these rates were affected by either GSH augmentation or GSH depletion. During normoxia in non-GSH-modified hearts, the amplitude of the FCR was 0.7 ± 0.02, and the amplitude of the SCR was 0.2 ± 0.03. This was not affected by GSH modification. After 25 min hypoxia, the amplitude of the FCR fell to 0.2 ± 0.03, while the amplitude of the SCR increased to 0.7 ± 0.05 (*p* < 0.05). These changes in respective amplitudes were not altered by modification of cardiac GSH status.

## Discussion

We have demonstrated that depletion or augmentation of GSH status to degrees matching or exceeding those measured in cardiovascular disease or cancer had no effect on the retention of ^64^Cu(ATSM) or its pharmacokinetic profile under either hypoxic or normoxic conditions. Furthermore, we have shown that inducing sufficient hypoxia to cause a significant increase in ^64^Cu trapping from ^64^Cu(ATSM) causes no measurable change in intracellular GSH status, at least in this acute experimental setting. Changes in intracellular GSH status within the limits of this study therefore do not appear to affect the hypoxia selectivity of ^64^Cu(ATSM), as had previously been suggested [10]. In 1972, Petering reported that thiols directly reduce Cu-KTS; however, little has since been reported on the GSH-mediated reduction of Cu(ATSM) [11]. GSH has more recently been demonstrated incapable of reducing Cu(ATSM) in an *in vitro* system [22]; however, this study did not preclude GSH acting as a cofactor in the (possibly enzymatic) reduction of the tracer inside the cell, nor did it replicate the relative concentrations of tracer and thiol that would occur *in vivo*; to quantify Cu(ATSM) spectrophotometrically, it was necessary for Xiao et al. to use concentrations of Cu(ATSM) many orders of magnitude higher than we used in our experiments, which are closer to those occurring *in vivo* during a PET scan. While BSO pre-treatment has previously been shown to not affect Cu(ATSM) retention in neuroblastoma cells under normoxic conditions, intracellular GSH concentrations in these experiments were not quantified, making it difficult to draw a definitive conclusion [23]. In our study, therefore, we employed a sensitive radiometric technique allowing us to investigate Cu(ATSM) pharmacokinetics at tracer concentrations in a physiologically relevant model over which we have the capacity to accurately modulate (and confirm) intracellular GSH concentrations.

Intracellular GSH status changes dramatically during many disease processes in response to increased oxidative stress. In the heart, atrial glutathione levels have been demonstrated to be 58% lower in NYHA class IV patients than in healthy subjects [14]. Experimentally, in isolated perfused rabbit hearts, the total GSH content fell by 54% after 90 min of ischaemia and by 61% 30 min after reperfusion. These degrees of GSH depletion are comparable to those we observe following BSO treatment in our model. The GSH content of cancer cells is, perhaps unsurprisingly, more variable. While adenocarcinomas and large cell carcinomas have been shown to have GSH levels approximately 27% lower than normal lung tissue, squamous cell carcinomas contain 207% of control values [24]. The drug resistance of many tumours is thought to be mediated in part by GSH levels elevated up to 50-fold greater than normal [25]–[27]. With such large variations in intracellular GSH status in these disease processes, which ^64^Cu(ATSM) purportedly targets by virtue of their hypoxic status, it is essential to confirm that this hypoxia selectivity is not affected by variations in intracellular GSH content. While our results suggest that changes in GSH concentration do not contribute to the hypoxia selectivity of ^64^Cu(ATSM), we are not proposing that ^64^Cu(ATSM) reduction is not GSH-mediated. While it is still possible that other reductants may be responsible for this process (and that they may change during hypoxia to cause increased tracer retention), the intracellular concentration of GSH in mammalian cells is millimolar, and in vast excess to the sub-nanomolar intracellular concentration of ^64^Cu(ATSM) present when injected as a PET tracer. As such, even the significant augmentation and depletion of intracellular GSH content that we achieve in our experiments does not impact meaningfully upon this ratio. Intracellular thiols remain likely candidates fundamental to the intracellular reduction of ^64^Cu(ATSM), but their high relative concentration means that GSH-mediated ^64^Cu(ATSM) reduction is not a rate-limiting factor in radiotracer retention. It is currently unclear whether the rate of reduction or dissociation may change during hypoxia, perhaps influenced by changes in intracellular pH [9],[10],[28], or whether the rate of tracer retention is purely governed by intracellular oxygen availability for reoxidation.

## Conclusion

Copper *bis*(thiosemicarbazone) complexes represent a versatile family of hypoxia imaging agents with a range of hypoxia selectivities for a variety of applications in both cardiology and oncology. To optimise the diagnostic and prognostic insight gained from the PET images with these complexes, it is essential to understand the nature of their tissue uptake and retention. Here, we demonstrate that their uptake is not sensitive to changes in intracellular GSH concentration within the range investigated and provide further confirmation of their specificity for tissue which is insufficiently oxygenated.

## Authors’ original submitted files for images

Below are the links to the authors’ original submitted files for images. Authors’ original file for figure 1 Authors’ original file for figure 2 Authors’ original file for figure 3 Authors’ original file for figure 4 Authors’ original file for figure 5 Authors’ original file for figure 6
