# Supplementary figures and images for: Modification of intracellular glutathione status does not change the cardiac trapping of 64Cu(ATSM)
Source: EJNMMI Res. 2014 Aug 1;4:40. doi: 10.1186/s13550-014-0040-8 (PMC4883992; doi:10.1186/s13550-014-0040-8)

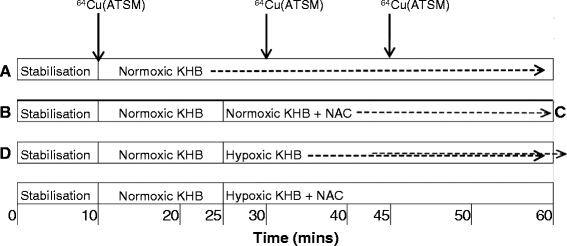

Supplement: Supplementary file 1 — Authors’ original file for figure 1 [file 13550_2014_40_MOESM1_ESM.gif]

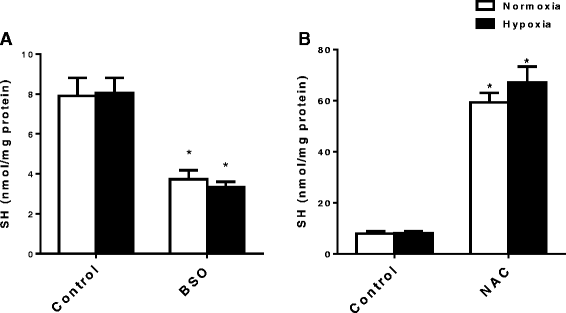

Supplement: Supplementary file 2 — Authors’ original file for figure 2 [file 13550_2014_40_MOESM2_ESM.gif]

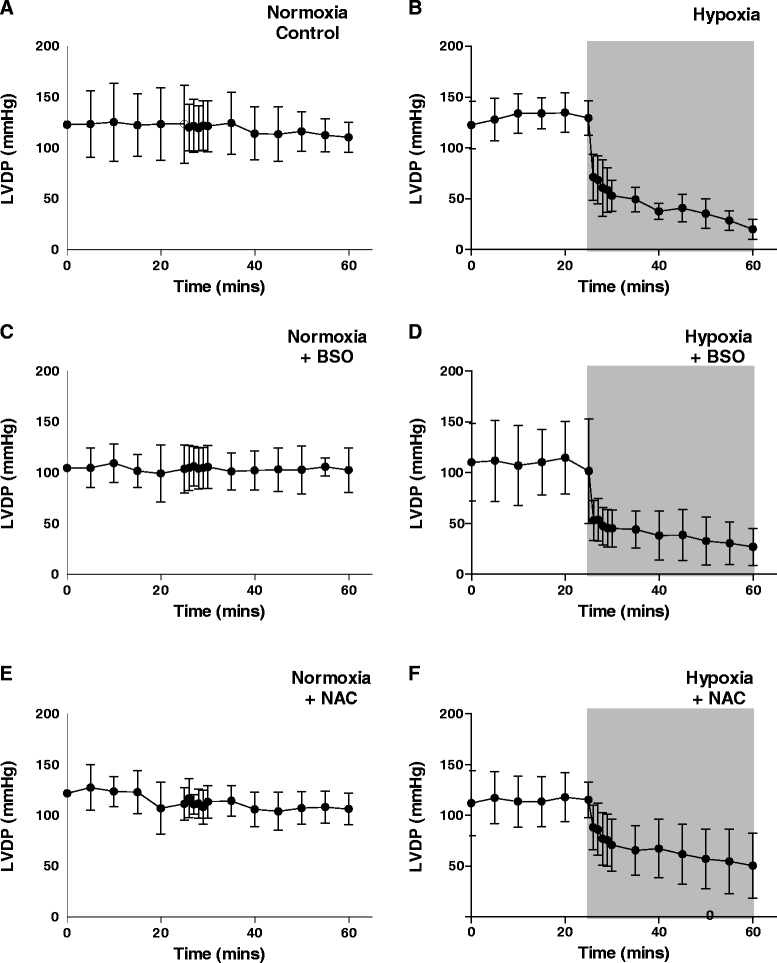

Supplement: Supplementary file 3 — Authors’ original file for figure 3 [file 13550_2014_40_MOESM3_ESM.gif]

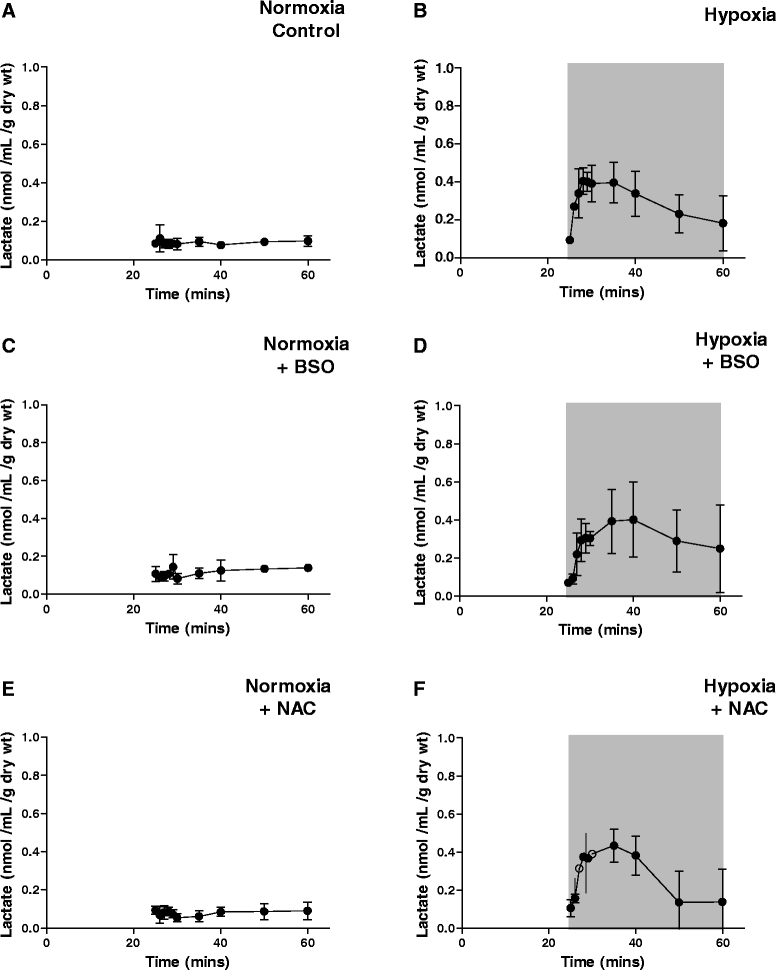

Supplement: Supplementary file 4 — Authors’ original file for figure 4 [file 13550_2014_40_MOESM4_ESM.gif]

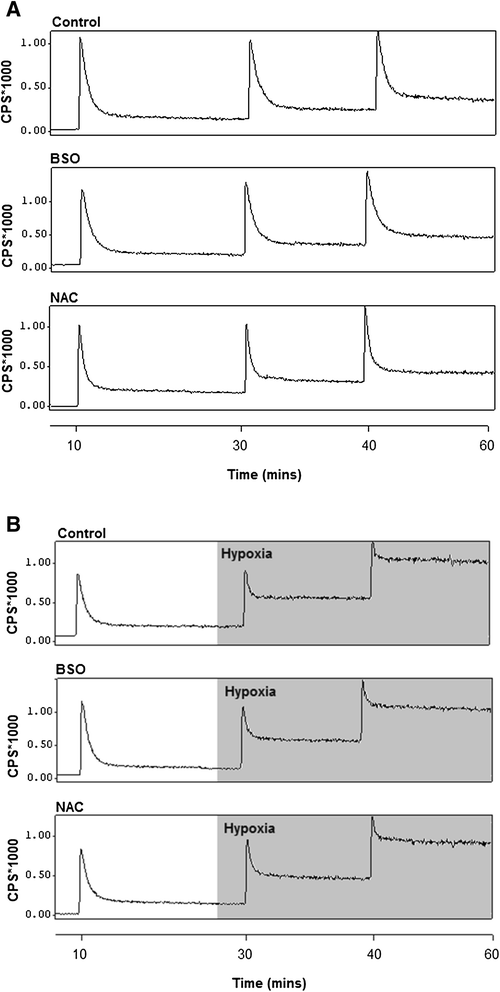

Supplement: Supplementary file 5 — Authors’ original file for figure 5 [file 13550_2014_40_MOESM5_ESM.gif]

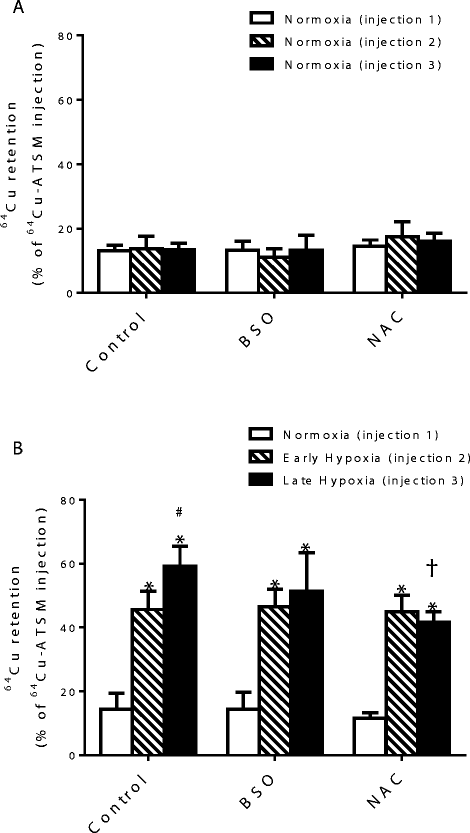

Supplement: Supplementary file 6 — Authors’ original file for figure 6 [file 13550_2014_40_MOESM6_ESM.gif]
